# Endocannabinoids in *Caenorhabditis elegans* are essential for the mobilization of cholesterol from internal reserves

**DOI:** 10.1038/s41598-018-24925-8

**Published:** 2018-04-23

**Authors:** Celina Galles, Gastón M. Prez, Sider Penkov, Sebastian Boland, Exequiel O. J. Porta, Silvia G. Altabe, Guillermo R. Labadie, Ulrike Schmidt, Hans-Joachim Knölker, Teymuras V. Kurzchalia, Diego de Mendoza

**Affiliations:** 1 Laboratorio de Fisiología Microbiana, Instituto de Biología Molecular y Celular de Rosario (IBR), CONICET, Facultad de Ciencias Bioquímicas y Farmacéuticas, Universidad Nacional de Rosario, 2000 Rosario, Argentina; 2 Max Planck Institute of Molecular Cell Biology and Genetics, 01307 Dresden, Germany; 3 Department of Genetics and Complex Diseases and Department of Cell Biology, Harvard T.H. Chan School of Public Health and Harvard Medical School, Boston, MA 02115 USA; 4 Instituto de Química Rosario (IQUIR), CONICET, Facultad de Ciencias Bioquímicas y Farmacéuticas, Universidad Nacional de Rosario, 2000 Rosario, Argentina; 5 Department Chemie, Technische Universität Dresden, Bergstr. 66, 01069 Dresden, Germany

## Abstract

Proper cholesterol transport is crucial for the functionality of cells. In *C*. *elegans*, certain cholesterol derivatives called dafachronic acids (DAs) govern the entry into diapause. In their absence, worms form a developmentally arrested dauer larva. Thus, cholesterol transport to appropriate places for DA biosynthesis warrants the reproductive growth. Recently, we discovered a novel class of glycosphingolipids, PEGCs, required for cholesterol mobilization/transport from internal storage pools. Here, we identify other components involved in this process. We found that strains lacking polyunsaturated fatty acids (PUFAs) undergo increased dauer arrest when grown without cholesterol. This correlates with the depletion of the PUFA-derived endocannabinoids 2-arachidonoyl glycerol and anandamide. Feeding of these endocannabinoids inhibits dauer formation caused by PUFAs deficiency or impaired cholesterol trafficking (e.g. in Niemann-Pick C1 or DAF-7/TGF-β mutants). Moreover, in parallel to PEGCs, endocannabinoids abolish the arrest induced by cholesterol depletion. These findings reveal an unsuspected function of endocannabinoids in cholesterol trafficking regulation.

## Introduction

Cholesterol is a lipid used by virtually all eukaryotic organisms. Its primary role as a structural component of cell membranes is to regulate their fluidity, permeability and topology. However, cholesterol also serves as a precursor of a plethora of other important biomolecules, such as steroid hormones, oxysterols and bile acids. Thus, tight regulation of the intake, biosynthesis, transport and metabolism of cholesterol is crucial for cellular integrity and functionality. In cells, cholesterol needs to be delivered to various destinations where it performs its structural functions or is further metabolized. This process is known as intracellular cholesterol trafficking and its importance is underscored by an amounting number of pathologies resulting from its impairment, including atherosclerosis and many genetic disorders such as the Niemann-Pick type C (NPC) disease and the Tangier disease^1,2^. Therefore, the cholesterol trafficking machinery has been intensively studied and many protein mediators of vesicular and non-vesicular cholesterol transport have been described^2^.

*C*. *elegans* is an excellent model for studying cholesterol trafficking and metabolism because worms are unable to synthesize cholesterol and require its presence in the diet. This allows an easy manipulation of worm sterol composition by introducing variations of its levels in the diet^3^. Following cholesterol intake, the systemic distribution of this lipid in worms is mediated by various transport systems, including the vitellogenin lipoprotein particles^4^. After receptor-mediated endocytosis of cholesterol carriers, cholesterol is trafficked through the endolysosomal system and directed to other cellular compartments with the assistance of the Niemann-Pick type C1 (NPC1) homologs NCR-1 and NCR-2^5^. The importance of this process is demonstrated by the fact that *ncr-2;ncr-1* null mutants fail to produce fertile adults and instead arrest at the dauer diapause, an alternative developmental stage for survival under harsh conditions such as overcrowding and starvation^5^. This developmental arrest has been shown to occur as a result of a decreased production of bile acid like steroid hormones called dafachronic acids (DAs)^5,6^. These important hormones integrate cues from various signaling pathways, including the transforming growth factor (TGF)-β-like (defined by the TGF-β homologue DAF-7) pathway, the insulin-like pathway (involving nematode insulin receptor DAF-2) and the cyclic GMP pathway, by binding a nuclear hormone receptor (NHR) named DAF-12^6–10^. In its DA-bound form, DAF-12 stimulates reproductive development, whereas in the absence of DAs it promotes dauer arrest.

Even though cholesterol is associated with cell membranes and interacts with multiple lipid species, very little is known about how lipids influence cholesterol trafficking. One of the few known examples is the positive effect of the phospholipid lysobisphosphatidic acid on the trafficking of cholesterol through the endolysosomal compartment^11^. Owing to the huge diversity of membrane lipids, multiple other lipid species might emerge as additional modulators of the cholesterol trafficking process. More recently, we have discovered a novel class of glycolipids, phosphoethanolamine glucosylceramides (PEGCs), that stimulates the trafficking of cholesterol in *C*. *elegans*^10^. PEGCs stimulate the growth of worms under conditions of cholesterol scarcity and abolish the developmental arrest of *ncr-2;ncr-1* mutants. By enhancing the mobilization of cholesterol from intracellular pools, PEGCs stimulate the production of DAs, thus inhibiting the dauer promoting activity of DAF-12.

Another class of lipids, endocannabinoids, have been implicated in the regulation of dauer formation as well^12^. These molecules are conserved lipid mediators that regulate multiple biological processes in a variety of organisms^13,14^. Previous studies have shown that one class of endocannabinoids, *N*-acylethanolamines (NAEs), antagonize dauer formation in *C*. *elegans*. NAEs are endocannabinoids composed of a fatty acyl chain of varying length and unsaturation (polyunsaturated fatty acids, PUFAs) linked to an ethanolamine head group. It has been shown that supplementation with the NAE eicosapentanoyl ethanolamide (EPEA), was able to rescue the dauer phenotypes of *daf-2* mutant lines^12^.

In this study, we show that the synthesis of PUFAs is important for cholesterol trafficking and, accordingly, for the reproductive development of worms. Further characterization of the bioactive PUFA-derivatives revealed that arachidonoyl-ethanolamine (AEA) and 2-*O*-arachidonoyl-glycerol (2-AG), the best-characterized arachidonic acid containing endocannabinoids^15^, are responsible for abolishing dauer larva arrest by stimulating cholesterol trafficking. We also show that endocannabinoids via mobilization of internal cholesterol pools can rescue the dauer arrest induced by PEGCs deficiency/cholesterol depletion. We propose that endocannabinoids and PEGCs act synergistically to promote growth and development by regulating cholesterol homeostasis in *C*. *elegans*.

## Results

### Depletion of PUFAs leads to enhanced dauer formation

**Figure 1:**
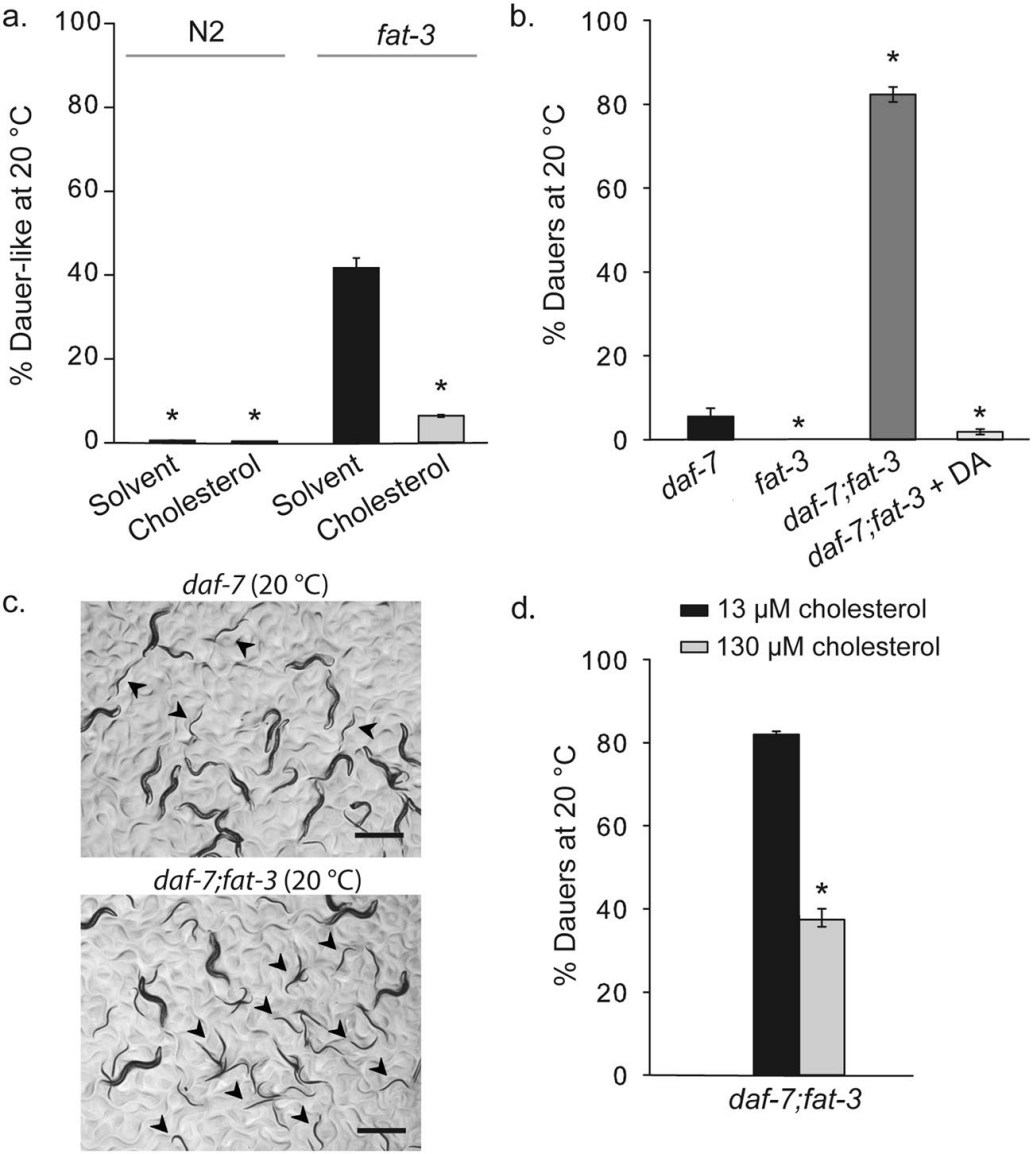
PUFA-deficient mutants *fat-3* and *daf-7;fat-3* exhibit a dauer-like or a Daf-c phenotype, respectively. (**a**) Unlike wild-type worms, *fat-3(ok1126)* mutants are incapable of synthesizing PUFAs and undergo a dauer-like arrest in the first generation when grown in cholesterol-free media. One-way analysis of variance p < 0.001. All pairwise multiple comparison procedures (Holm-Sidak method), (*) indicates statistically significant difference with *fat-3*/solvent, p < 0.001. Bars represent mean values and error bars represent standard errors. The number of independent experiments is n = 3 for all conditions. **(b)** While *daf-7* worms form ~10% dauers at 20 ºC, *fat-3* worms do not form dauers. In stark contrast, double mutant *daf-7;fat-3* forms ~85% dauers at 20 ºC, and addition of DA suppresses dauer formation almost completely. One-way analysis of variance p < 0.001. All pairwise multiple comparison procedures (Holm-Sidak method): (*) indicates statistically significant difference with *daf-7*, p < 0.001. Bars represent mean values and error bars represent standard errors. The number of independent experiments is n = 5 for *daf-7*, and n = 13 for *fat-3* and *daf-7; fat-3*. [DA] = 90 nM. **(c)** Introducing the *fat-3* null-mutation into background *daf-7* increases dauer formation significantly (dauers are indicated with arrowheads). Representative images are taken from at least three experiments. Scale bars, 0.5 mm. **(d)** *daf-7;fat-3* dauers can partly bypass dauer arrest if grown in excess cholesterol (130 µM). (*) indicates statistically significant difference to control plates (13 µM cholesterol). t-test, p < 0.001. Bars represent mean values and error bars represent standard errors. The number of independent experiments is n = 14 for *daf-7*;*fat-3* at 13 µM cholesterol, and n = 2 for *daf-7;fat-3* at 130 µM cholesterol.

In a previous study, we have shown that *C*. *elegans* interrupts reproductive development and arrests as a dauer-like larva (L2*) with incomplete molting when grown for two generations without cholesterol^3^. More recently, we have found that this arrest can be abolished by supplementation of PEGCs, substances belonging to a novel class of lipids that mobilizes internal pools of cholesterol^10^. We were interested to further investigate this process and to identify other components that might be involved in it. We reasoned that strains aberrant in cholesterol mobilization would arrest already in the first generation without externally provided sterols. Indeed, a small screening performed on a mutant strain collection (Mende, F. and Kurzchalia, T.V., unpublished data) indicated that one of them, with inactive Δ^6^-desaturase [*fat-3(ok1126)*] showed growth defects already in the first, rather than in the second generation when grown without cholesterol. Unlike the wild type strain, that produced almost 100% gravid adults in the first generation without cholesterol, *fat-3* displayed a high incidence of arrested larvae (Fig. 1a) with typical dauer morphology (Supplementary Figure 1). This mutant strain bears a large deletion in the coding region of gene *fat-3*, and is null for FAT-3 desaturase. Animals accumulate linoleic and α-linolenic acids and are unable to produce the major C20 PUFAs found in *C*. *elegans*: arachidonic (20:4 (n-6), AA) and eicosapentanoic acid (20:5(n-3), EPA)^16,17^.

Next, we set out to confirm the interaction between the PUFA biosynthesis pathways and the dauer formation regulating pathways. In particular, we tested the interaction with the DAF-7/TGF-β pathway that regulates dauer development by affecting sterol trafficking and metabolism^10,18,19^. In our previous study we have shown that temperature-sensitive *daf-7* mutants with “dauer formation constitutive” (Daf-c) phenotype are hypersensitive to cholesterol depletion and form dauer larvae in the absence of external cholesterol already at the semi-permissive growth temperature despite internally stored sterols^10^. We reasoned that if the biosynthesis of PUFAs is connected with this regulatory pathway, depletion of PUFAs should enhance the phenotype of *daf-7*. To this end, we generated a double mutant strain for *fat-3(ok1126)* and *daf-7(e1372)* and scored dauer formation. When grown at the semi-permissive growth temperature (20° C), *daf-7(e1372);fat-3(ok1126)* double mutants formed significantly more dauers (~85%) than the parental *daf-7* strain (<10%) (Fig. 1b,c). Moreover, enhanced dauer formation in double mutants was fully abolished when the medium was supplemented with DA (Fig. 1b). These results suggest that a decrease in the synthesis of C20 PUFAs could be leading to deficiency of DAs. We speculated that depletion of PUFAs might exacerbate cholesterol deficiency due to a decrease in its delivery rate from storage sites to the locations of DA production. Therefore, we tested whether very high cholesterol availability in the growth medium was sufficient to compensate for the loss of FAT-3 in *daf-7;fat-3* animals. As shown in Fig. 1d, providing a high-cholesterol diet did promote dauer recovery of *daf-7;fat-3* mutants.

### Arachidonic acid-containing endocannabinoids abolish dauer formation of *daf-7;fat-3* and *daf-7;fat-4* mutants

**Figure 2:**
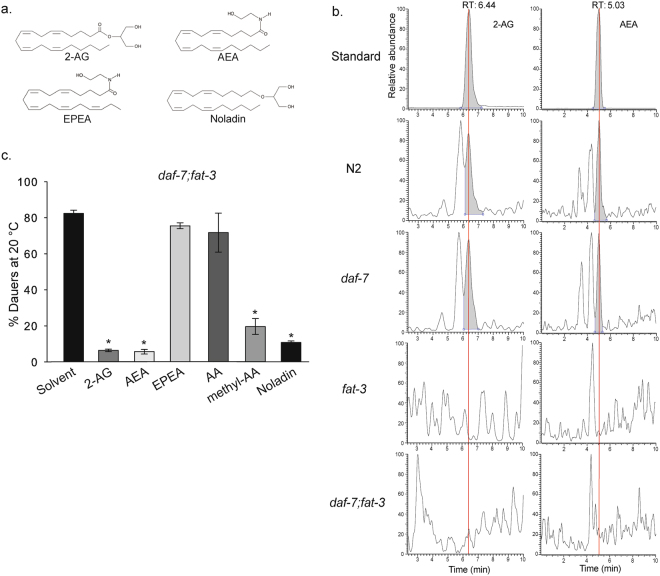
Endocannabinoids rescue the Daf-c phenotype of *daf-7;fat-3* worms. (**a**) Chemical structures of the endocannabinoid molecules assayed. **(b)** Extracted ion chromatograms of HPLC- MS/MS experiments in order to detect the endocannabinoids 2-AG and AEA in lipid extracts of the following worm strains: N2, *daf-7*, *fat-3* and *daf-7;fat-3*. The upper panels show the chromatograms of the analytical standards 2-AG and AEA. **(c)** *daf-7;fat-3* dauers cannot be rescued by supplementation with EPEA. However, addition of the endocannabinoids 2-AG and AEA, and the non-hydrolysable noladin ether suppresses dauer formation significantly. Supplementation of methyl-AA also prevents *daf-7;fat-3* dauer formation. Kruskal-Wallis one-way analysis of variance on ranks, p < 0.001. Multiple comparisons versus control group (Dunn’s Method). (*) indicates statistically significant difference with solvent, p < 0.05. Bars represent mean values and error bars represent standard errors. The number of independent experiments is n = 14 for solvent, n = 2 for EPEA and noladin, n = 14 for 2-AG, n = 4 for AEA and AA, and n = 8 for methyl-AA. The concentration of all additives is 50 µM.

Then, we asked which PUFA-derived molecules could be responsible for the dauer arrest of *daf-7;fat-3* double mutants. PUFAs are metabolized into many potent bioactive compounds such as eicosanoids and endocannabinoids^20^. The latter, shown in Fig. 2a, might be one of the most likely class of candidates because previous studies have shown that *N*-acylethanolamines (NAEs) antagonize dauer formation in *C*. *elegans*^12^. Using high-performance liquid chromatography coupled to mass spectrometry (HPLC MS/MS), we found that, in agreement with a previous report^21^, N2 has detectable amounts of 2*O*-arachidonoylglycerol (2-AG, Fig. 2a) and arachidonoyl-ethalonamine (anandamide or AEA, Fig. 2a) (Fig. 2b and Supplementary Table 1). We also determined that these arachidonic acid-containing endocannabinoids are synthesized by *daf-7* (Fig. 2b). In contrast, both *fat-3* and *daf-7;fat-3* strains do not synthesize PUFA-derived endocannabinoids to detectable levels (Fig. 2b). Next, we tested the capability of the detected endocannabinoid molecules to suppress dauer formation of *daf-7;fat-3*. We found that supplementation of the media with 2-AG and AEA significantly reduced the Daf-c phenotype of *daf-7;fat-3* worms (Fig. 2c). In contrast, EPEA failed to rescue the Daf-c phenotype of *daf-7;fat-3* (Fig. 2a,c).Treatment with AA, which is both a precursor as well as the hydrolytic breakdown product of both 2-AG and AEA^15^, was unable to promote reproductive growth of *daf-7;fat-3* (Fig. 2c). However, in a previous study, we have established the use of fatty acid methyl esters (FAMEs) instead of the corresponding free fatty acids due to the much higher rescue activities of the former^22^. FAMEs are more active presumably because membranes are more permeable for them. Indeed, addition of AA-methyl ester (methyl-AA) to *daf-7;fat-3* worms significantly reduced the Daf-c phenotype (Fig. 2c). This raised the question whether methyl-AA is a precursor of 2-AG and/or AEA^23^ or AA has an effect by itself. In order to find out whether methyl-AA serves as a precursor of 2-AG and AEA, lipid extracts of *fat-3* and *daf-7;fat-3* mutants fed with methyl-AA were analyzed by LC-MS/MS. This analysis revealed that indeed these mutants were capable to synthesize both 2-AG and AEA (Supplementary Table 1) upon methyl-AA supplementation. Importantly, we found that noladin^24^ (Fig. 2a), a non-hydrolysable analog of 2-AG, was also able to rescue the Daf-c phenotype of *daf-7;fat-3* to an extent comparable to the effect of 2-AG, demonstrating that the endocannabinoids are not merely precursors for other AA-derived compounds (Fig. 2c).

Although AA-derived endocannabinoids rescue the dauer phenotype of *daf-7;fat-3*, it could be argued that this double mutant is unable to synthesize dihommo-γ-linolenic acid [20:3(n-6)] (DGLA) and eicosatetraenoic acid [20:4(n-3)], [ETA (n-3)] and that endocannabinoids derived from these C20 PUFAs could serve the same function as 2-AG or AEA. To answer this question we generated *daf-7;fat-4* double mutant strain that synthesizes DGLA and ETA (n-3), but is unable to synthesize AA^25^ due to its deficiency in Δ5 fatty acid desaturase enzyme FAT-4. *daf-7;fat-4* double mutant displays a very strong Daf-c phenotype similar to the one observed in *daf-7;fat-3* (Supplementary Figure 2a). Furthermore, supplementation of the growth medium with an excess of cholesterol, DA, 2-AG and noladin ether substantially lowers dauer formation in *daf-7;fat-4* (Supplementary Figures 2a and 2b). These results demonstrate the specificity of AA-derived endocannabinoids to rescue the enhanced dauer formation of *daf-7* animals deprived of C20 PUFAs. Taken together, we conclude that endocannabinods containing AA residues are the active compounds promoting reproductive development.

### Endocannabinoids do not substitute for DA

As shown above, both endocannabinoids and DAs are able to rescue the dauer constitutive phenotype displayed by *daf-7;fat-3* (Figs 1b and 2c). This raised the possibility that the endocannabinoid compounds were merely substituting for DAs as DAF-12 ligands. To address this question, we tested whether 2-AG and AEA were able to compensate for the deficiency of DAF-9 cytochrome P450 enzyme, necessary for the production of DA from cholesterol^6,26^. As seen in Supplementary Figure 3, unlike DA, 2-AG and AEA failed to inhibit dauer formation in *daf-9(dh6)* mutants. This indicates that these endocannabinoids cannot substitute for DA. Thus, it seems likely that these endocannabinoids inhibit dauer formation by enhancing the synthesis of DAs.

### Endocannabinoids rescue the arrest induced by cholesterol depletion through its mobilization

**Figure 3:**
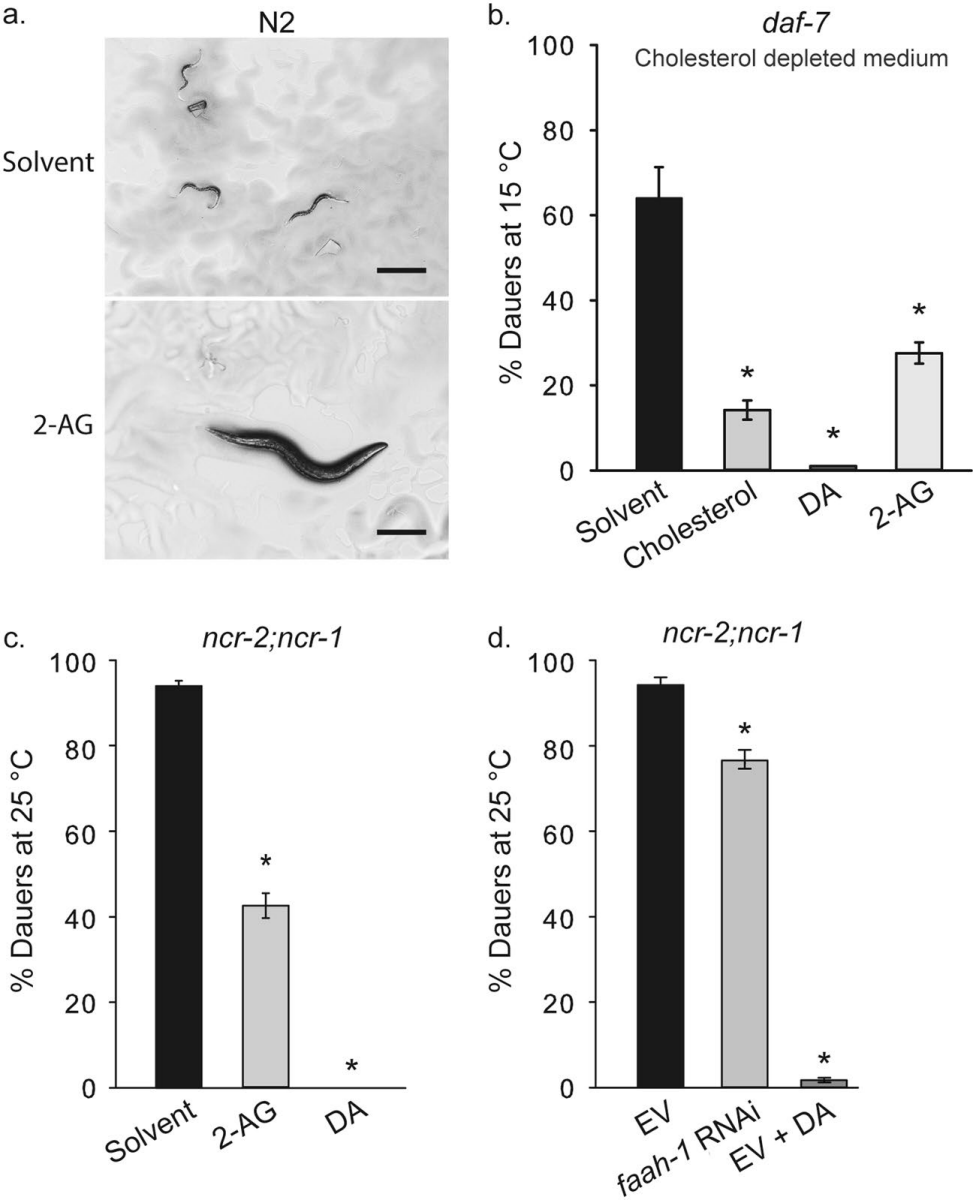
Endocannabinoids suppress dauer formation induced by cholesterol depletion. (**a**) Wild-type control animals grown for two generations in the absence of cholesterol arrest as L2-like larvae (upper micrograph), however, when fed with 2-AG worms form adults (lower micrograph). Representative images from at least three experiments. Scale bars, 0.25 mm. **(b)** When grown in the absence of cholesterol *daf-7* worms form ~65% dauers in the first generation at 15 ºC. This arrest is largely suppressed by the addition of either cholesterol, or DA, or by the endocannabinoid 2-AG. (*) indicates statistically significant difference with solvent control. t-test, p = 0.002. Bars represent mean values and error bars represent standard errors. The number of independent experiments is n = 5 for solvent, and 2-AG, n = 7 for cholesterol, and n = 4 for ∆^7^-DA. [Cholesterol] = 13 µM. [DA] = 90 nM. [2-AG] = 50 µM. (**c)** 2-AG suppresses the Daf-c phenotype of *ncr-2;ncr-1* at 25 ºC. (*) indicates statistically significant difference with solvent control. Kruskal-Wallis one-way analysis of variance on ranks, p < 0.001 and all pairwise multiple comparison procedures (Student-Newman-Keuls Method), p < 0.05. Bars represent mean values and error bars represent standard errors. The number of independent experiments is n = 6 for all conditions. [Cholesterol] = 13 µM. [DA] = 90 nM. [2-AG] = 50 µM. **(d)** *faah-1* RNAi reduces the percent of dauer formation of strain *ncr-2;ncr-1* at 25 ºC. (*) indicates statistically significant difference with solvent empty vector control (EV). One Way Analysis of Variance (p < 0.001) and comparison by Holm-Sidak method, p < 0.001 in both cases. Bars represent mean values and error bars represent standard errors. The number of independent experiments is n = 6 for all conditions tested. [DA] = 90 nM.

Our previous studies have demonstrated that PEGCs also enhance the synthesis of DAs by mobilization of internal pools of maternally deployed cholesterol^10^. Because of this activity, PEGCs are also able to abolish the developmental arrest caused by cholesterol starvation in the second generation of worms grown on sterol-depleted medium^10^. We asked whether endocannabinoids could similarly inhibit the arrest of cholesterol-depleted worms. Indeed, addition of 2-AG to sterol-lacking medium resulted in formation of many late stage (L4) larvae and adults in the N2 wild-type strain in the second generation, whereas the untreated worms arrested mainly as L2* larvae as published before (Fig. 3a)^3^.

We next set out to investigate whether AEA and 2-AG have the ability to mobilize internal reserves of sterols in *C*. *elegans*. First, we made use of *daf-7* mutants which are hypersensitive to cholesterol starvation. They form dauers already in the first generation even at permissive temperature (15˚C) when the external cholesterol source is missing. Although these worms receive high amounts of maternal cholesterol, they are unable to use this sterol for DA synthesis presumably due to an impairment in mobilizing sterols efficiently^10^. We found that upon cholesterol deprivation, 2-AG rescues the dauer arrest of *daf-7* animals grown without cholesterol (Fig. 3b). Next, we investigated the interaction of endocannabinoids with the NPC1 sterol-trafficking system^27^. As mentioned earlier, cholesterol transport in *C*. *elegans* is partly mediated by the NPC1 homologs NCR-1 and NCR-2^28^. Because of this, double mutant *ncr-2(nr2023);ncr-1(nr2022)* displays constitutive dauer formation^5^. This dauer formation can be rescued by supplementation with DAs^5,7^. As seen in Fig. 3c, 2-AG could also largely suppress *ncr-2;ncr-1* dauer formation. Moreover, the Daf-c phenotype of *ncr-2;ncr-1*, as well as of *daf-7*, was diminished by RNAi against *faah-1*, a gene encoding a conserved fatty acid amide hydrolase (FAAH) enzyme, responsible for the breakdown of *N*-acylethanolamines such as AEA and EPEA in worms^12^ (Fig. 3d and Supplementary Figure 4).

The ability of the AA-derived endocannabinoids to rescue the development of cholesterol-depleted worms pointed out to the possibility that the endogenous production of these compounds might be enhanced in response to cholesterol withdrawal. This could be a mechanism to optimize cholesterol mobilization when the diet is not rich in sterols. In agreement with this hypothesis, we found that removing cholesterol from the growth medium resulted in a reproducible two-fold increase in 2-AG levels in N2 animals already in the first generation (Supplementary Table 2). Taken together, our data suggest that in *C*. *elegans* endocannabinoids exert their effects on post-embryonic development by enhancing the efficiency of cholesterol trafficking and the utilization of internal pools.

### Endocannabinoids and PEGCs are acting through parallel pathways

Next, we examined how 2-AG and AEA are connected to recently discovered PEGCs: Can endocannabinoids rescue PEGCs deficiency? And *vice versa*, can PEGCs rescue endocannabinoid deficiency?

**Figure 4:**
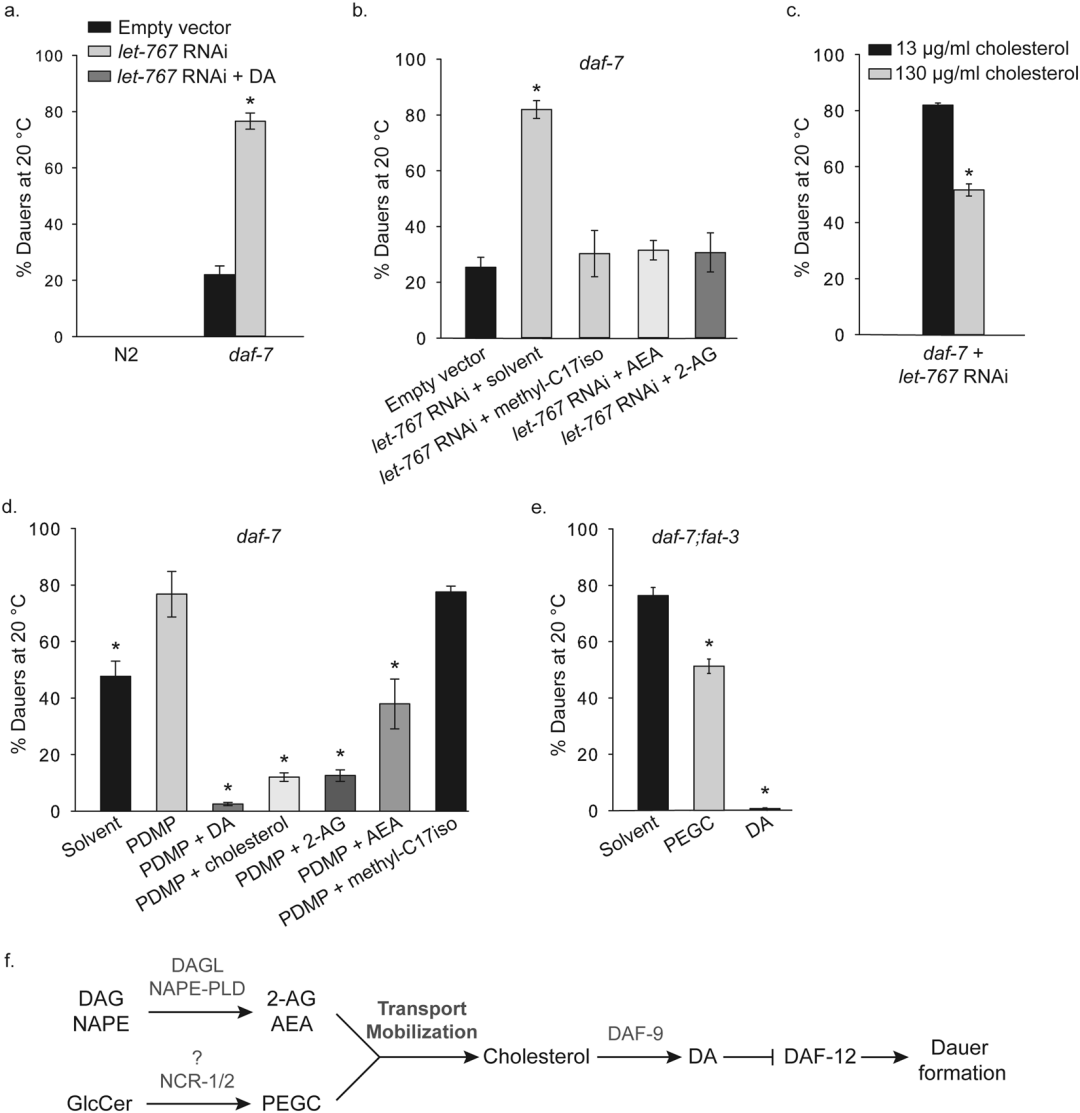
PEGCs and endocannabinoids stimulate cholesterol transport by employing parallel pathways. (**a**) RNAi of *let-767* leads to a significant increase of dauers compared to control plates in *daf-7* mutant background (empty vector; from ~20% to ~80%), but has no effect on wild type (N2) dauer formation. Kruskal-Wallis one-way analysis of variance on ranks, p < 0.001, All pairwise multiple comparison procedures (Dunn’s Method), (*) indicates statistically significant difference between *daf-7*/empty vector and *daf-7*/*let-767* RNAi, p < 0.05. Bars represent mean values and error bars represent standard errors. The number of independent experiments is n = 6 for N2/empty vector, and N2/*let-767* RNAi, n = 27 for *daf-7*/empty vector, n = 33 for *daf-7*/*let-767* RNAi, and n = 6 for *daf-7*/*let-767* RNAi+DA. [DA] = 90 nM. **(b)** *daf-7* on *let-767* RNAi at 20 ºC forms ~80% dauers. Supplementation with either of the endocannabinoids 2-AG and AEA, or the addition of methyl-C17iso suppresses dauer formation significantly. Kruskal-Wallis one-way analysis of variance on ranks, p < 0.001. Multiple comparisons versus control group (Dunn’s Method). (*) indicates statistically significant difference with empty vector, p < 0.05. Bars represent mean values from at least three independent experiments and error bars represent standard errors. [2-AG] or [AEA] = 50 µM. [methyl-C17iso] = 100 µM. **(c)** *daf-7 let-767* RNAi worms partly bypass dauer arrest if grown in excess cholesterol (130 µM). (*) indicates statistically significant difference with control plates (13 µM cholesterol). Mann-Whitney rank sum test p = 0.013. Bars represent mean values and error bars represent standard errors. The number of independent experiments is n=26 for *daf-7 let-767* RNAi at 13 µM cholesterol, and n = 4 for *daf-7 let-767* RNAi at 130 µM cholesterol. **(d)** Inhibition of the d17iso-glucosylceramide synthesis (and hence PEGCs) by PDMP treatment leads to an increase in dauer formation in *daf-7* background, that can be largely suppressed by endocannabinoids, DA and excess cholesterol, but not by methyl-C17iso. One-way analysis of variance, p < 0.001. Multiple comparisons versus control group (Holm-Sidak method). (*) indicates statistically significant difference with PDMP treatment, p < 0.001 for all conditions except for empty vector (p = 0.002). Bars represent mean values and error bars represent standard errors. The number of independent experiments is n = 3 for all conditions. [DA] = 90 nM. [Cholesterol] = 130 µM. [2-AG] or [AEA] = 50 µM. [methyl-C17iso] = 100 µM. **(e)** mmPEGC-C22 is partly able to suppress dauer formation of *daf-7;fat-3* at 20 ºC. One-way analysis of variance, p < 0.001. Multiple comparisons versus control group (Holm-Sidak method). (*) indicates statistically significant difference with solvent control, p < 0.001 for both conditions. Bars represent mean values and error bars represent standard errors. The number of independent experiments is n = 2 for all conditions. [DA] = 1 µM. [mmPEGC-C22] = 0.5 mM. **(f)** Endocannabinoids and PEGCs act in parallel to promote transport/mobilization of cholesterol from internal pools. Endocannabinoids are synthesized from their precursors DAG and NAPE most probably by DAGL and NAPE-PLD enzymatic activities. The enzyme(s) that produce(s) PEGC from d17iso-GlcCer is(are) unknown but the synthesis is dependent on NCR-1/2. Both endocannabinoids and PEGCs have the ability to mobilize cholesterol. The latter is then delivered to the sites of DAF-9 mediated DA synthesis. After systemic distribution of DA within the organism, it binds and inhibits the dauer-promoting activities of DAF-12. DAG – diacylglycerol; NAPE - *N*-acyl-phosphatidylethanolamine; DAGL - DAG lipase; NAPE-PLD - NAPE-specific phospholipase D.

To induce PEGCs deficiency, we inhibited the synthesis of their precursors, the glucosylceramides (GlcCer). For this, two approaches have been used. Firstly, we decided to decrease C15iso and C17iso monomethyl branched-chain fatty acids (mmBCFA), needed for the synthesis of the long-chain base in *C*. *elegans*^29^ (Supplementary Figures 5 and 6). For this, we applied RNAi of LET-767, the major 3-ketoacyl-CoA reductase, necessary for the elongation of long-chain and branched-chain fatty acids. RNAi of LET-767 in wild type worms leads to multiple developmental phenotypes in the first generation and early larval arrest in the second generation (L1-L2)^22^. Indeed, RNAi against LET-767 in *daf-7* mutants grown at 20 °C significantly increases the Daf-c phenotype of the strain (Fig. 4a). GC-MS analysis of the relative fatty acid profiles of control and *let-767* RNAi animals confirmed that RNAi treatment leads to a strong decrease in the levels of C15iso and C17iso mmBCFAs, but not of PUFAs (Supplementary Table 3). Accordingly, as seen in Fig. 4b, supplementation with C17iso mmBCFA methyl esters rescued the *let-767* RNAi-induced dauer arrest of *daf-7* worms. Astoundingly, both 2-AG and AEA also rescued the dauer formation observed in *daf-7* animals subjected to *let-767* RNAi (Fig. 4b**)**. To exclude the possibility that endocannabinoids act by elevating levels of mmBCFAs, i.e. by inducing (a) not yet identified alternative 3-ketoacyl-CoA reductase(s), we performed GC-MS analysis of mmBCFA in *daf-7 let-767* RNAi worms in the presence of 2-AG (Supplementary Table 4). This experiment clearly shows that endocannabinoid treatment does not increase mmBCFA. In consistency with impaired cholesterol trafficking in *daf-7 let-767* (RNAi), dauer formation was partly inhibited by high cholesterol levels in the medium (Fig. 4c).

The second approach to deplete PEGCs was to treat *daf-7* with PDMP, an inhibitor of the final glucosylation step in the synthesis of GlcCer (Supplementary Figure 6). We found that DA, high cholesterol and endocannabinoids rescue *daf-7* dauers generated in the presence of PDMP (Fig. 4d)^10^. However, as expected, PDMP treatment could not be rescued by addition of mmBCFA C17iso methyl ester because PDMP inhibits a later step of the synthesis of PEGCs. Thus, PEGCs deficiency could be rescued by supplementation with endocannabinoids.

In another set of experiments, we investigated whether PEGCs could abolish dauer formation in the endocannabinoids deficient strain *daf-7;fat-3*. As seen in Fig. 4e, synthetic mmPEGC-C22, the major PEGC species found in worms^10^, can partially rescue the dauer formation induced by the absence of endocannabinoids. Taking altogether, we propose that two unrelated lipid species, endocannabinoids and PEGCs, regulate worm cholesterol transport/mobilization through parallel pathways.

## Discussion

The major finding of our study is that the endocannabinoids 2-AG and AEA regulate the transport/mobilization of cholesterol in *C*. *elegans*, which results in enhanced DA production. Several lines of evidence support this notion: endocannabinoids (i) abolish the developmental arrest caused by cholesterol depletion in wild-type and *daf-7* worms, (ii) stimulate the reproductive development of *daf-7* worms with diminished glucosylceramide/PEGC synthesis and (iii) rescue *ncr-2;ncr-1* mutants with aberrant cholesterol transport.

Based on our data, we propose a tentative scheme of the interaction between endocannabinoids and PEGC in the control of cholesterol transport (Fig. 4f). Multiple reactions produce the precursors diacylglycerol (DAG), *N*-acyl-phosphatidylethanolamine (NAPE), and GlcCer required for the synthesis of 2-AG, AEA and PEGCs, respectively. The conversions of these precursors to the active compounds are carried out by not yet identified enzymes in *C*. *elegans*. 2-AG and AEA are probably produced by enzymes homologous to the mammalian DAG lipase (DAGL) and NAPE-specific phospholipase D (NAPE-PLD)^30^, although alternative biosynthetic pathways also exist^12,20,31^. The enzymatic system required for PEGCs production is completely unknown. Intriguingly, PEGCs cannot be synthesized in the absence of functional NCR-1 and NCR-2 proteins^10^. This suggests that in worms PEGCs mediate cholesterol transport downstream of NPC1. Endocannabinoids and PEGCs act in parallel to stimulate the mobilization/transport of cholesterol. This leads to delivery of the latter to DAF-9, production of DA and inhibition of the dauer-promoting activity of DAF-12. It should be noted that, in addition to their effect as regulators of cholesterol mobilization, endocannabinoids might also stimulate the synthesis of DA via regulation of alternative signaling pathways such as the monoaminergic neurotransmission, GPCR and MAP kinase – mediated cascades, etc.^32,33^. PEGCs, on the other hand, could also display pleiotropic effects due to their specific glycospingolipid structure: they might physically interact with cholesterol or affect its distribution by regulating different properties of the cellular membranes: their topology, fluidity or permeability^34^.

One important implication of the fact that the endocannabinoids and GlcCer/PEGCs act in parallel is that endocannabinoids might be involved in the regulation of a plethora of other known glucosylceramide-dependent processes in worms. These include the activation of the nutrient-sensing TOR signaling, the establishment of epithelial polarity in intestinal cells, foraging behavior and sensory neuron maturation^29,35–37^. Future studies will reveal the relationships between glycosphingolipids, cholesterol and endocannabinoids in the control of these processes.

Many of the physiological effects produced by endocannabinoids are not completely understood. Some of them may reflect their influence on cholesterol trafficking. In higher organisms, many aspects of the intracellular cholesterol transport are regulated by the transcription factors LXR and SREBP^1^. Intriguingly, LXR is one of the vertebrate homologs of DAF-12, which binds oxysterols^38^. This points to the interesting possibility that LXR and DAF-12 might have similar endocannabinoid-dependent activities in regard to the control of cholesterol homeostasis. On the other hand, endocannabinoids and endocannabinoid agonists are known to increase the hepatic expression of SREBP in mice^39^. Whether 2-AG and AEA could also operate via SREBP in worms is worth investigating.

Finally, deregulation of cholesterol and lipid homeostasis has major consequences on development and disease. Cholesterol metabolism in particular is closely interrelated with cardiovascular disease in humans. It is well known that dietary supplementation with omega-6 polyunsaturated fatty acids (PUFAs) reduces the risk of cardiovascular disease. In part, this is attributable to the observation that increased plasma levels of omega-6 PUFAs favorably affect plasma LDL-cholesterol and HDL-cholesterol levels^40,41^. While it is widely accepted that lipid metabolism is linked to cholesterol homeostasis^38,42^, exactly how PUFAs control cholesterol balance and metabolism remains largely unexplored. Hence, our findings point up the exciting scenario that the role of PUFA-derived endocannabinoids could be conserved in humans. Thus, unraveling the mechanisms by which endocannabinoids affect sterol-regulated processes in *C*. *elegans* could have important implications for the better understanding of human pathological conditions associated with impaired cholesterol homeostasis, such as atherosclerosis, non-alcoholic fatty liver disease and Niemann-Pick type C disease.

## Materials and Methods

### Materials

2-AG, AEA, noladin, EPEA and DL-*threo*-PDMP were purchased from Cayman Chemical (Ann Arbor, Michigan, USA). Cholesterol, AA, C17iso fatty acid, Dubelcco´s medium (DMEM) and antioxidant BHT were purchased from Sigma (Sigma-Aldrich, St. Louis, Missouri, USA). mmPEGC-C22^10^, Δ^4^-DA and Δ^7^-DA were provided by Prof. H.-J. Knölker^43–45^.

The worm strain N2 Bristol and the single mutants *daf-7(1372)*, *fat-3(ok1126)*, *fat-4 (ok958)*, *ncr-1;ncr-2* (JT10800) were obtained from Caenorhabditis Genetics Center (CGC). *daf-9(dh6);dhEx24* was provided by Prof. A. Antebi.

### Growth and maintenance of worm strains

Worms were routinely propagated on nematode growth medium (NGM) agar plates seeded with *E*. *coli* OP50^46^. Saturated cultures of *E*. *coli* OP50 or NA22 grown in lysogeny broth (LB) medium were concentrated 10 times and spread on NGM-agar plates. Worms were placed on the plates either as mixed stage populations or as embryos obtained by hypochlorite treatment. The temperature-sensitive Daf-c mutants were grown at 15 °C to grow in the reproductive mode and at 25 °C to arrest as dauer larvae.

### RNAi by feeding

RNAi against *let-767* was performed with *E*. *coli* HT115 double-stranded RNA (dsRNA)-producing strain from the Ahringer library. RNAi was performed in the first generation. Bleached embryos were left to hatch overnight in M9 at 20 °C and the resulting L1s were seeded to RNAi plates and grown at 20 °C or 15 °C according to the experiment. Similarly, *faah-1* RNAi was performed in the first generation. Unlike the experiments with *let-767*, the worms subjected to *faah-1* RNAi were seeded on the dsRNA-producing *E*. *coli* as embryos. Finally, the dauer phenotype was scored after 72 h or 144 h, depending on the assay. For biochemical analysis worms were harvested from RNAi plates after 72 h.

### Dauer formation assays

In general, 80–100 L1s or embryos were transferred to NGM plates seeded with *E*. *coli* (HT115) containing RNAi against *let-767*, *faah-1* or empty vector as a negative control. The supplementation with endocannabinoids (final concentration 50 µM), arachidonic acid (final concentration 50 µM), methyl esters from different fatty acids (final concentration 50 µM), mm-PEGC-C22 (final concentration 10 µM) and DL-threo-PDMP (final concentration 20 µM) was added to the bacteria immediately prior to seeding. The final concentrations of these compounds were calculated according to the volume of the NGM agar used for the preparation of the plates. It should be noted that the active concentrations of mm-PEGC-C22 published before^10^ have been calculated according to the volume of the bacterial loan seeded on the agar/agarose surface. If calculated using the latter method, the concentration of mm-PEGC-C22 used here would be 500 µM. Finally, after 72 h the percentage of dauers was scored. Δ^4^-DA and Δ^7^-DA^43,44^ were used alternatively in dauer rescue experiments with working concentrations that were chosen (1 µM and 90nM, respectively).

To determine dauer formation of *daf-9(dh6)* strain, embryos were placed on NGM plates with *E*. *coli* complemented with Δ^7^-DA or endocannabinoids. *dhEx24* is an extrachromosomal array carrying active DAF-9 and nuclear GFP. After 3 days *daf-9* null worms were identified on the basis of the absence of GFP signal and dauer formation was scored^47^.

### Cholesterol deprivation assay

The protocol from Matyash *et al*.^3^ was carried out. Briefly, to produce sterol-depleted media, agar was replaced by agarose (Sigma-Aldrich) that was washed three times with chloroform to deplete the trace sterols in it. *E*. *coli* NA22 was grown in low-glucose, sterol-free DMEM culture medium (Sigma-Aldrich). Bacteria were rinsed with M9 before use and concentrated 20 times. Then the plates were seeded with 100 µl of bacteria and the supplements were added (or solvent carrier as negative control). 80–100 L1s were plated, incubated at 15° C for 6 days, and finally scored for percentage of dauers formed.

### Lipid extraction and endocannabinoid analysis by HPLC-MS/MS

The protocol is an adaptation from Folch (1957)^48^. Lipid extracts were generated from approximately 100 mg of frozen worm pellets belonging to N2, *daf-7*, *fat-3* and *daf-7;fat-3* grown at 20 °C. The pellets were thawed on ice in 1.3 ml methanol and subjected to 4 min sonication on ice. After sonication, 1000 ppb of the internal standard 2-AG-d5 were added followed by 2.6 ml chloroform and 1.3 ml 0.5 M KCl/0.08 M H_3_PO_4_ to a final ratio of 1:2:1. Samples were vortexed and then sonicated in an ultrasonic water bath for 15 min. After vortexing twice for 1 min, samples were centrifuged for 10 min at 2.000 *g* to induce phase separation. The lower phase was collected into a clean glass tube, dried under nitrogen and re-suspended in 100 µl of acetonitrile.

2-AG and AEA were quantified from nematode samples by liquid chromatography (Ultimate 3000 RSLC Dionex, Thermo Scientic) coupled with an ESI triple quadrupole mass spectrometer (TSQ Quantum Access Max (QQQ), Thermo-Scientific). Reversed-phase HPLC column (C18 Hypersil-GOLD (50 x 2.1 mm), Thermo Scientific) was carried out employing the following step-wise gradient: 0 min (water 4:6 acetonitrile); 0.5 min (water 4:6 acetonitrile); 6.5 (water 2.5:7.5 acetonitrile); 7.5 min (water 2.5:7.5 acetonitrile); 8 min (water 4:6 acetonitrile). Column temperature was maintained at 40 °C and autosampler tray temperature was set at 10 °C. The following ionization conditions were used: ESI, positive-ion mode; drying gas (N_2_) temp., 300 °C; drying gas flow rate, 10 l/min; nebulizer pressure, 10 UA, and cap. voltage, 4 kV. Analyte detection was performed using NRM with the following transitions: m/z 379,2→289,2 for 2-AG; m/z 348,2→62,2 for AEA and m/z 384,2→289,2 for 2-AG-d5. Deuterated internal standard 2-AG-d5 was used for quantification, and peak-area ratios of the analyte to the internal standard were calculated as a function of the concentration ratios of the analyte to the internal standard. Sample concentrations were calculated from the calibration curve using an equally weighted linear regression (X) algorithm, where X represents concentration.

### Generation of *daf-7;fat-3* and *daf-7;fat-4* double mutant strains

Homozygous *fat-3(ok1126)* male worms were crossed to *daf-7(e1372)* hermaphrodites. The hermaphrodites from the progeny laid eggs that at 25 °C developed into dauers or adults. The dauers were relocated to 15 °C to re-enter reproduction and their progeny was screened using PCR for homozygous *daf-7(e1372);fat-3(ok1126)* individuals. Using an equivalent strategy, homozygous *fat-4(ok958)* male worms were crossed to *daf-7(e1372)* hermaphrodites. The hermaphrodites from the progeny laid eggs that at 25 °C developed into dauers or adults. The dauers were relocated to 15 °C to re-enter reproduction and their progeny was screened using PCR for homozygous *daf-7(e1372);fat-4(ok958)* individuals.

### GC-MS determination of fatty acid methyl-ester content

Lipid extraction was performed using Bligh and Dyer^49^ method. Briefly, the N2 and *fat-3* were grown at 20 °C on *E*. *coli* OP50 for 72 h and then harvested from plates with 5–10 ml of M9 buffer. For the interference assay with *let-767* RNAi, *daf-7* worms were grown on *E*. *coli* OP50 with TH115 empty vector as a control, and with RNAi against *let-767* at 20 °C for 72 h. Subsequently, worms were harvested as described above. Thereafter, worms were washed three times with M9 buffer to eliminate excess bacteria. The worm pellet was transferred to a glass tube and 3 ml of a chloroform:methanol 1:2 mixture was added. Dibutylhydroxytoluene (BHT) was added as an antioxidant at a final concentration of 5 µg/ml. The samples were incubated for 12 h at −20 °C. After incubation the samples were centrifuged and the supernatants were transferred to a new tube. 1 ml of KCl (1M) and 1 ml of chloroform were added to the samples, the samples were mixed by vortexing and then centrifugated for 2 min at 2000 *g*. Finally, the organic phases were transferred to new tubes and dried under N_2_ flow.

### Chemical General Information

^1^H and ^13^C NMR spectra were acquired on a Bruker Avance II 300 MHz (75.13 MHz) using CDCl_3_ as solvent. Electrospray Ionization High-Resolution Mass Spectra (ESI-HRMS) were recorded on a Bruker MicrOTOF II with lock spray source. IR spectra were obtained using an FTIR Shimadzu spectrometer. Chemical reagents were purchased from commercial suppliers and used without further purification, unless otherwise noted. Solvents were analytical grade or were purified by standard procedures prior to use. Yields were calculated for material judged homogeneous by thin layer chromatography (TLC) and nuclear magnetic resonance (^1^H-NMR). All reactions were monitored by thin layer chromatography performed on silica gel 60 F254 pre-coated aluminum sheets, visualized by a 254 nm UV lamp, and stained with an ethanolic solution of 4-anisaldehyde. Column flash chromatography was performed using silica gel 60 (230–400 mesh).

### 2-AG-d_5_ preparation

For obtaining 2-AG-d_5_ as a deuterated internal standard for quantification assays, we followed a three-step protocol: differential protection, esterification and deprotection.

Differential protection step: In order to only protect primary alcohols, a solution of glycerol-d_8_ (38 mg, 0.18 mmol) in anhydrous dichloromethane (5 ml) under inert atmosphere was prepared and cooled at −78 ºC. Then, collidine (54 mg, 0.36 mmol) and *tert*-butyl dimethyl silyl chloride (TBDMSCl, 70 mg, 0.54 mmol) was added. The mixture reacted for 3 h at −78 ºC, then warmed to room temperature and stirred for additional 12 h. Brine was added and the solution was extracted with dichloromethane. The combined organic extracts were dried over sodium sulfate and evaporated. Products were purified by column chromatography on silica gel with increasing hexane/ethyl acetate gradients. 1-*O*,3-*O*-Bis(TBDMS) glycerol-d_6_ was obtained as a colorless liquid in 44% yield (55 mg). For the esterification step, a solution of 1-*O*,3-*O*-Bis(TBDMS) glycerol-d_6_ (10 mg, 0.03 mmol) in anhydrous dichloromethane (2 ml) under inert atmosphere was prepared and cooled at 0 ºC. Next, arachidonic acid (36 mg, 0.12 mmol), 4-dimethylaminopyridine (15 mg, 0.12 mmol) and *N*,*N*’-dicyclohexylcarbodiimide (15 mg, 0.12 mmol) were added in that order. The mixture reacted for 3 h at 0 ºC, and was then warmed to room temperature and stirred for additional 12 h. Water was added and the solution was extracted with dichloromethane. Combined organic extracts were dried over sodium sulfate and evaporated. Products were purified by column chromatography on silica gel with increasing hexane/ethyl acetate gradients. 1-*O*,3-*O*-bis(TBDMS)−2-AG-d_5_ was obtained as a yellowish liquid in 80% yield (15 mg). For the deprotection step, a solution of 1*O*,3*O*-Bis(TBDMS)−2-AG-d_5_ (15 mg, 0.025 mmol) in tetrahydrofuran (2 ml) was prepared. Then, a 1M solution of tetrabutylammonium fluoride (150 ml, 0.15 mmol) was added cautiously dropwise. Once the reaction was complete (1 h), water was added and the solution was extracted with dichloromethane. Combined organic extracts were dried over sodium sulfate and evaporated. A quantitative amount of product 2-AG-d_5_ (10 mg, quantitative) was obtained as a yellowish liquid.

### Methyl- ester preparation

For the derivatization of fatty acids prior to GC-MS analysis, 0.5 ml of anhydrous sodium methoxide 0.5 M were added to the lipid samples and then incubated 30 min at room temperature. Then 1 ml of HCl (2 N) was added for neutralization of the reaction. The fatty acid methyl esters were extracted with hexane and finally resuspended in 500 ml of hexane.

For obtaining the AA and C17iso methyl-esters used for supplementation assays we followed a protocol based on Walker *et al*.^50^. Briefly, a solution of diazomethane in diethyl ether was added cautiously dropwise to a stirred solution of the appropriate fatty acid (50 mg, 0.16 mmol) in dichloromethane (5 ml) until the yellow color persisted. The excess of the reagent was vented under a nitrogen purge. The solvent was evaporated and the product dried under vacuum to provide the methyl ester as a colorless oil (54 mg, quantitative) that was used without further purification.

### Gas chromatography coupled to mass spectrometry (GC-MS)

The methyl ester analysis was performed in a Shimadzu GC-2010 Plus equipment. The column used was a Supelco WAX-10 (Sigma Aldrich) 100% polyethyleneglycol. The helium flux was 1 ml/min and the heating program was 180 °C (0 min to 32 min) and then a gradient increasing 3 °C/min from 180 °C to 240 °C. The split was 1/30 and the ionization voltage was 70 eV with an ionic range from 50 to 600 Da. The ion specters were registered as relative abundance in function of mass/charge (*m/z*). Peak assignation was done using the mixture of standards of fatty acid methyl esters AGs Supelco (Sigma Aldrich).

## Electronic supplementary material

Supplementary Information

## References

[1] Ikonen E (2008). Cellular cholesterol trafficking and compartmentalization. Nat. Rev. Mol. Cell Biol..

[2] Iaea DB, Maxfield FR (2015). Cholesterol trafficking and distribution. Essays Biochem..

[3] Matyash V (2004). Sterol-derived hormone(s) controls entry into diapause in *Caenorhabditis elegans* by consecutive activation of DAF-12 and DAF-16. PLoS Biol..

[4] Matyash V (2001). Distribution and transport of cholesterol in *Caenorhabditis elegans*. Mol. Biol. Cell.

[5] Li J, Brown G, Ailion M, Lee S, Thomas JH (2004). NCR-1 and NCR-2, the *C*. *elegans* homologs of the human Niemann-Pick type C1 disease protein, function upstream of DAF-9 in the dauer formation pathways. Development.

[6] Motola DL (2006). Identification of ligands for DAF-12 that govern dauer formation and reproduction in *C*. *elegans*. Cell.

[7] Fielenbach N, Antebi A (2008). *C*. *elegans* dauer formation and the molecular basis of plasticity. Genes Dev..

[8] Antebi A, Yeh WH, Tait D, Hedgecock EM, Riddle DL (2000). *daf-12* encodes a nuclear receptor that regulates the dauer diapause and developmental age in *C*. *elegans*. Genes Dev..

[9] Antebi A, Culotti JG, Hedgecock E (1998). M. daf-12 regulates developmental age and the dauer alternative in *Caenorhabditis elegans*. Development.

[10] Boland S (2017). Phosphorylated glycosphingolipids essential for cholesterol mobilization in *Caenorhabditis elegans*. Nat. Chem. Biol..

[11] Kobayashi T (1999). Late endosomal membranes rich in lysobisphosphatidic acid regulate cholesterol transport. Nat. Cell Biol..

[12] Lucanic M (2011). N-acylethanolamine signalling mediates the effect of diet on lifespan in *Caenorhabditis elegans*. Nature.

[13] Skaper SD, Di Marzo V (2012). Endocannabinoids in nervous system health and disease: the big picture in a nutshell. Philos. Trans. R. Soc. Lond. B. Biol. Sci..

[14] McPartland JM, Matias, Marzo I, Di V, Glass (2006). M. Evolutionary origins of the endocannabinoid system. Gene.

[15] Iannotti FA, Di Marzo V, Petrosino S (2016). Endocannabinoids and endocannabinoid-related mediators: Targets, metabolism and role in neurological disorders. Prog. Lipid Res..

[16] Watts JL, Browse J (2002). Genetic dissection of polyunsaturated fatty acid synthesis in Caenorhabditis elegans. Proc. Natl. Acad. Sci. USA.

[17] Watts JL, Phillips E, Griffing KR, Browse J (2003). Developmental Defects in Caenorhabditis elegans fat-3 Mutants..

[18] Ren P (1996). Control of *C*. *elegans* larval development by neuronal expression of a TGF-beta homolog. Science.

[19] Liu T, Zimmerman KK, Patterson GI (2004). Regulation of signaling genes by TGF-beta during entry into dauer diapause in *C*. *elegans*. BMC Dev. Biol..

[20] Vrablik TL, Watts JL (2013). Polyunsaturated fatty acid derived signaling in reproduction and development: insights from *Caenorhabditis elegans* and *Drosophila melanogaster*. Mol. Reprod. Dev..

[21] Lehtonen M (2011). Determination of endocannabinoids in nematodes and human brain tissue by liquid chromatography electrospray ionization tandem mass spectrometry. J. Chromatogr. B.

[22] Entchev EV (2008). LET-767 is required for the production of branched chain and long chain fatty acids in *Caenorhabditis elegans*. J. Biol. Chem..

[23] Silvestri C, Di Marzo V (2013). The endocannabinoid system in energy homeostasis and the etiopathology of metabolic disorders. Cell Metab..

[24] Hanus L (2001). 2-arachidonyl glyceryl ether, an endogenous agonist of the cannabinoid CB1 receptor. Proc. Natl. Acad. Sci. USA.

[25] Watts JL, Ristow M (2017). Lipid and carbohydrate metabolism in Caenorhabditis elegans. Genetics.

[26] Gerisch B (2007). A bile acid-like steroid modulates *Caenorhabditis elegans* lifespan through nuclear receptor signaling. Proc. Natl. Acad. Sci. USA.

[27] Rosenbaum AI, Maxfield FR (2011). Niemann-Pick type C disease: Molecular mechanisms and potential therapeutic approaches. J. Neurochem..

[28] Sym M, Basson M, Johnson C (2000). A model for Niemann – Pick type C disease in the nematode *Caenorhabditis elegans*. Curr. Biol..

[29] Zhu H, Shen H, Sewell AK, Kniazeva M, Han M (2013). A novel sphingolipid-TORC1 pathway critically promotes postembryonic development in *Caenorhabditis elegans*. Elife.

[30] Harrison N (2014). Characterization of N-Acyl Phospholipase-D Isoforms in the Nematode Caenorhabditis elegans. PLoS One.

[31] Lin Y-CY-H (2014). Diacylglycerol lipase regulates lifespan and oxidative stress response by inversely modulating TOR signaling in Drosophila and C. elegans. Aging Cell.

[32] Pastuhov SI, Matsumoto K, Hisamoto N (2016). Endocannabinoid signaling regulates regenerative axon navigation in *Caenorhabditis elegans* via the GPCRs NPR-19 and NPR-32. Genes to Cells.

[33] Oakes, M., Jing Law, W., Clark, T., Bamber, B. & Komuniecki, R. Cannabinoids activate monoaminergic signaling to modulate key *C*. *elegans* behaviors. *J*. *Neurosci*. 10.1523/JNEUROSCI.3151-16.2017 (2017). 10.1523/JNEUROSCI.3151-16.2017 PMC5354331 28188220

[34] Hannich JT, Umebayashi K, Riezman H (2011). Distribution and Functions of Sterols and Sphingolipids. Cold Spring Harb. Perspect. Biol..

[35] Zhang H (2011). Apicobasal domain identities of expanding tubular membranes depend on glycosphingolipid biosynthesis. Nat. Cell Biol..

[36] Kniazeva, M., Crawford, Q. T., Seiber, M., Wang, C. Y. & Han, M. Monomethyl branched-chain fatty acids play an essential role in *Caenorhabditis elegans* development. *PLoS Biol*. **2** (2004). 10.1371/journal.pbio.0020257 PMC514883 15340492

[37] Kniazeva M, Zhu H, Sewell AK, Han M (2015). A Lipid-TORC1 Pathway Promotes Neuronal Development and Foraging Behavior under Both Fed and Fasted Conditions in *C*. *elegans*. Dev. Cell.

[38] Hong C, Tontonoz P (2014). Liver X receptors in lipid metabolism: opportunities for drug discovery. Nat. Rev. Drug Discov..

[39] Osei-hyiaman D (2005). Endocannabinoid activation at hepatic CB 1 receptors stimulates fatty acid synthesis and contributes to diet-induced obesity. J. Clin. Invistigation.

[40] Harris WS (2009). Omega-6 fatty acids and risk for cardiovascular disease: A science advisory from the American Heart Association nutrition subcommittee of the council on nutrition, physical activity, and metabolism; council on cardiovascular nursing; and council on epidem. Circulation.

[41] Katan MBB (2009). Omega-6 polyunsaturated fatty acids and coronary heart disease. Am. J. Clin. Nutr..

[42] Lee SD, Tontonoz P (2015). Liver X receptors at the intersection of lipid metabolism and atherogenesis. Atherosclerosis.

[43] Martin R (2009). Synthesis and biological activity of the (25R)-cholesten-26-oic acids-ligands for the hormonal receptor DAF-12 in Caenorhabditis elegans. Org. Biomol. Chem..

[44] Martin R, Däbritz F, Entchev EV, Kurzchalia TV, Knölker H-J (2008). Stereoselective synthesis of the hormonally active (25S)-D7-dafachronic acid, (25S)-D4-dafachronic acid and (25S)-Cholestenoic Acid. Org. Biomol. Chem..

[45] Saini R (2012). Stereoselective synthesis and hormonal activity of novel dafachronic acids and naturally occurring steroids isolated from corals. Org. Biol. Chem..

[46] Brenner S (1974). The Genetics of *Caenorhabditis elegans*. Genetics.

[47] Gerisch B, Weitzel C, Kober-eisermann C, Rottiers V, Antebi A (2001). A Hormonal Signaling Pathway Influencing *C*. *elegans* Metabolism, Reproductive Development, and Life Span. Dev. Cell.

[48] Folch, J., Lees, M. & Sloane Stanley, G. H. A Simple method for the Isolation and Purification of Total Lipids from Animal Tissues. **55**, 999–1033 (1987). 13428781

[49] Bligh EG, Dyer WJ (1959). A Rapid Method of Total Lipid Extraction and Purification. Can. J. Biochem. Physiol..

[50] Walker M, Roberts D, Dumbroff EB (1982). Convenient apparatus for methylating small samples with diazomethane. J. Chromatogr..

