# Supplementary material for: Endocannabinoids in Caenorhabditis elegans are essential for the mobilization of cholesterol from internal reserves
Source: Sci Rep. 2018 Apr 23;8:6398. doi: 10.1038/s41598-018-24925-8 (PMC5913221; doi:10.1038/s41598-018-24925-8)
Supplement: Supplementary file 1 — Supplementary Information [file 41598_2018_24925_MOESM1_ESM.pdf]

Endocannabinoids in *Caenorhabditis elegans* are essential for the mobilization of cholesterol from internal reserves

Galles, Celina[a#], Prez, Gastón M.[a#], Penkov, Sider[b#], Boland, Sebastian[c], Porta, Exequiel O. J.[d], Altabe, Silvia G.[a], Labadie, Guillermo R.[c], Schmidt, Ulrike[e], Knölker, Hans-Joachim[e], Kurzchalia, Teymuras V.[b*] and de Mendoza, Diego[a*]

[a]Laboratorio de Fisiología Microbiana, Instituto de Biología Molecular y Celular de Rosario (IBR), CONICET, Facultad de Ciencias Bioquímicas y Farmacéuticas, Universidad Nacional de Rosario, 2000, Rosario, Argentina.

[b]Max Planck Institute of Molecular Cell Biology and Genetics, 01307 Dresden, Germany.

[c]Department of Genetics and Complex Diseases, Harvard T.H. Chan School of Public Health, Boston, MA 02115, U.S.A.

[d]Instituto de Química Rosario (IQUIR), CONICET, Facultad de Ciencias Bioquímicas y Farmacéuticas, Universidad Nacional de Rosario 2000, Rosario, Argentina.

[e]Department Chemie, Technische Universität Dresden, Bergstr. 66, 01069 Dresden, Germany.

[#]These authors contributed equally to this work

*For correspondence: demendoza@ibr-conicet.gov.ar or kurzchalia@mpi-cbg.de

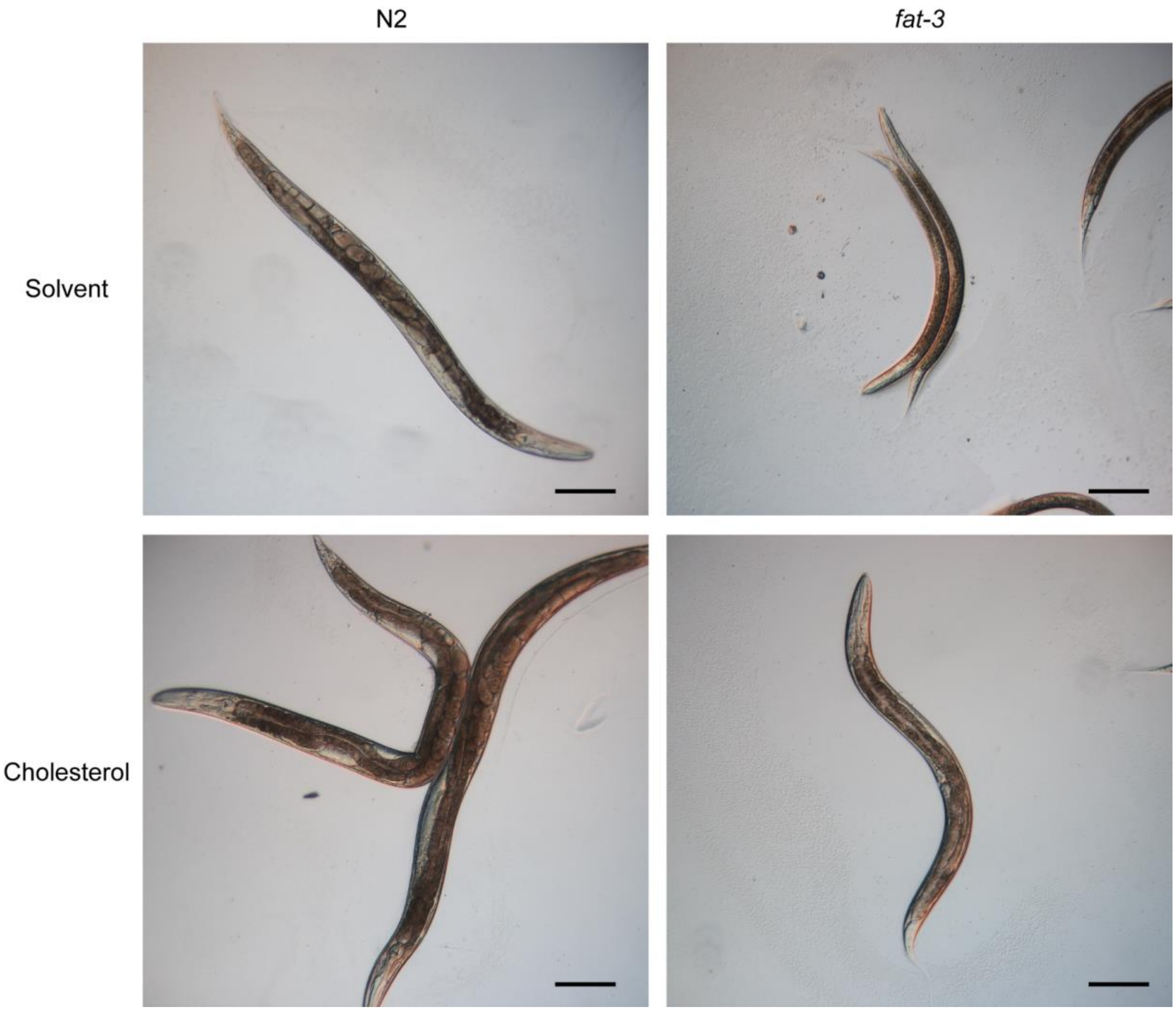

Supplementary Figure 1. Sterol-depletion leads to dauer-like morphology in *fat-3* mutants. N2 worms grown in cholesterol-depleted media reach adulthood (upper left panel), while *fat-3* worms mostly arrest as dauer-like animals (upper right panel). Both strains exclusively form adults in the presence of cholesterol (lower panels). Representative images from at least three experiments. Nomarski microscopy. Scale bars, 0.1 mm.

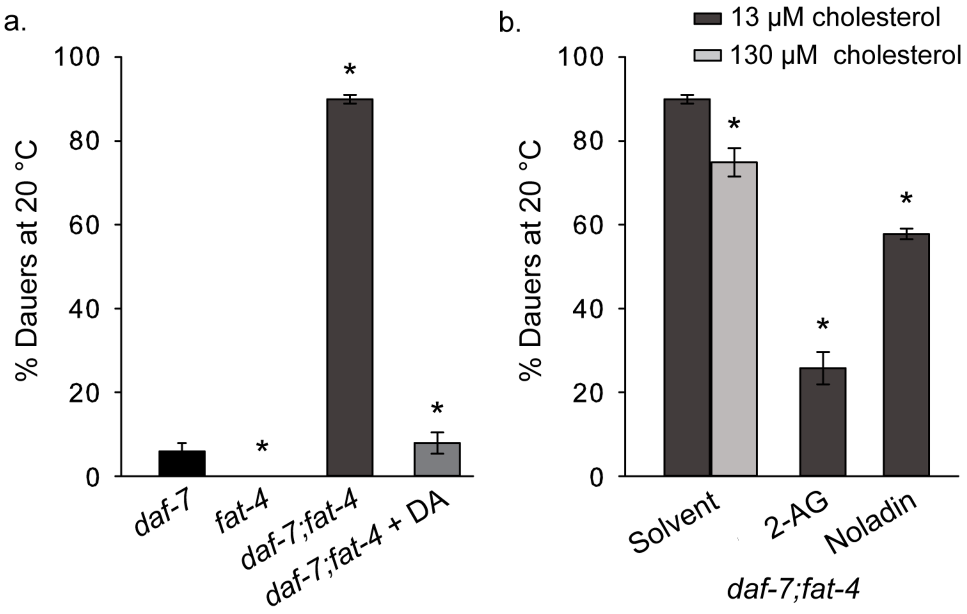

Supplementary Figure 2. Arachidonic acid-deficient mutants *daf-7;fat-4* exhibit a Daf-c phenotype at 20 ºC, which can be overcome by endocannabinoid supplementation. (a) As opposed to *daf-7* and *fat-4* strains, double mutant *daf-7;fat-4* forms ~85% dauers at 20 ºC, and addition of DA suppresses dauer formation almost completely. One-way analysis of variance, $p<0.001$. (*) indicates statistically significant difference with *daf-7,* multiple comparison by Holm-Sidak method, $p<0.05$ for all conditions. Bars represent mean values and error bars represent standard errors. The number of independent experiments is n=3 for all conditions. [DA]=90 nM. **(b)** Addition of excess cholesterol, 2-AG and noladin effectively reduces dauer formation in each case. One-way analysis of variance $p<0.001$. (*) indicates statistically significant difference with cholesterol 13 µM , multiple comparison by Holm-Sidak method, $p<0.05$ for cholesterol 130 µM and $p<0.001$ for the remaining conditions. Bars represent mean values and error bars represent standard errors. The number of independent experiments is n=3 for all conditions, except for 2-AG where n=9. [DA]=90 nM. [2-AG]=50 µM. [Noladin]=100 µM.

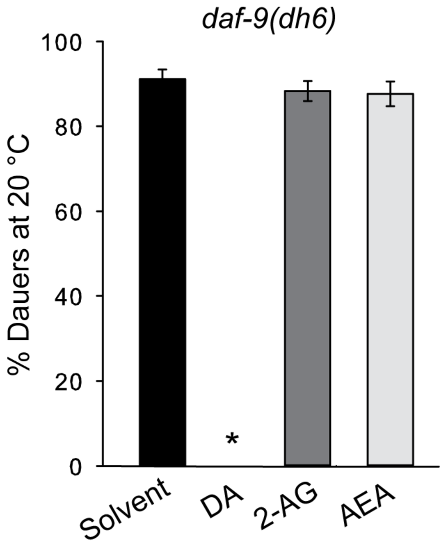

Supplementary Figure 3. Endocannabinoids cannot substitute for dafachronic acid. *daf-9*(*dh6*) mutant worms are unable to produce DAs, and hence form dauer larvae. Supplementing the food with DA suppresses this arrest. However, addition of endocannabinoids 2-AG and AEA does not suppress this phenotype. Kruskal-Wallis One Way Analysis of Variance on Ranks, $p<0.001$, All Pairwise Multiple Comparison Procedures (Tukey's Method), (*) indicates statistically significant difference with control (solvent) $p<0.05$. Bars represent mean values and error bars represent Standard Errors. The amount of independent experiments is n=6 for all conditions. [DA] = 90 nM. [2-AG] or [AEA] = 50 µM.

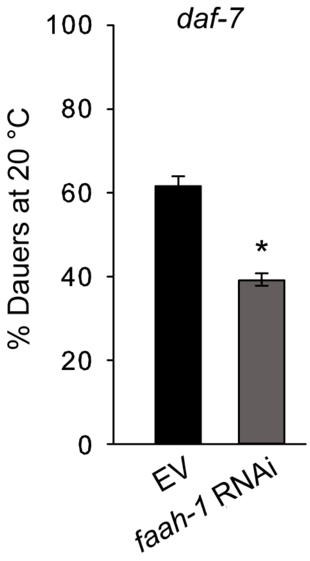

Supplementary Figure 4. RNAi for *faah-1* effectively reduces the Daf-c phenotype of *daf-7* strain at 20 ºC. t-test, $p<0.001$. (*) indicates statistically significant difference with empty vector control (EV). Bars represent mean values and error bars represent Standard Errors. The amount of independent experiments is n=6 for both conditions.

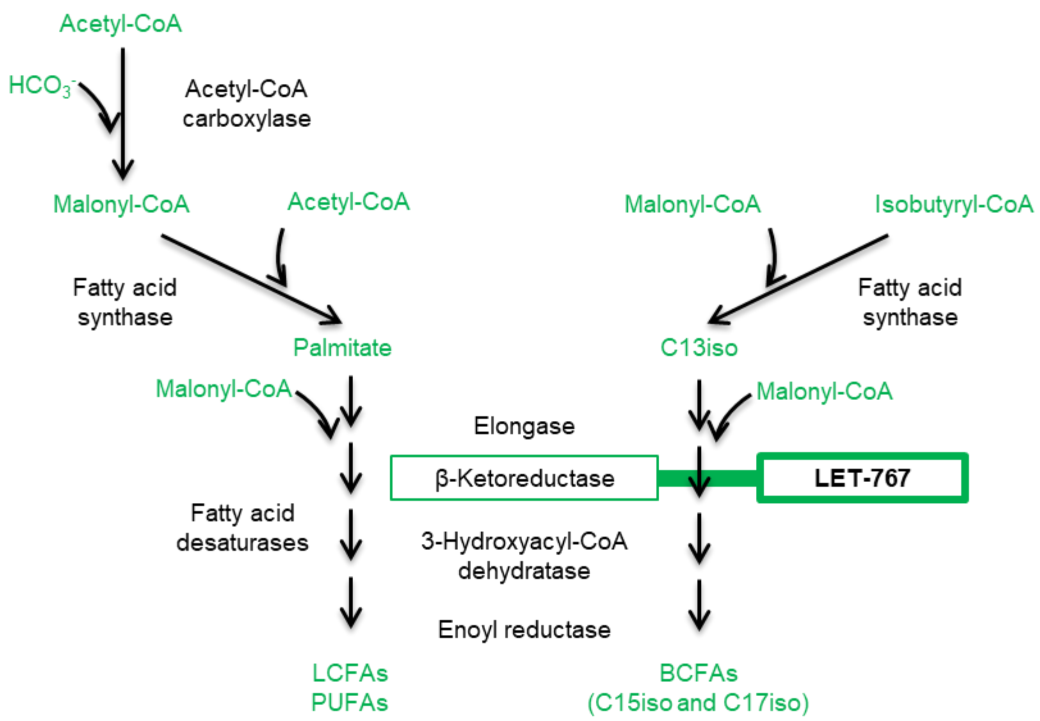

Supplementary Figure 5. Fatty acid synthesis and elongation in *C. elegans*. *C. elegans* is capable of synthesizing long-chain fatty acids from either acetyl-CoA precursors or dietary palmitate. Acetyl-CoA is condensed with HCO_3^- to generate malonyl-CoA by the acetyl-CoA carboxylase enzyme. Malonyl-CoA consecutively incorporates two carbon units for its elongation up to palmitate by the enzyme fatty acid synthase. Several elongases, LET-767 3-ketoacyl-CoA-reductase, 3-hydoxyacyl-CoA dehydratase and enoyl reductase activities and seven desaturases (FAT-1 through FAT-7) are necessary for LCFA remodeling and PUFA synthesis. The synthesis from Isobutyryl-CoA to C13iso is also performed by fatty acid synthase enzyme. Then, C13iso is further elongated to longer BCFAs (C15iso and C17iso) by the action of elongase enzymes ELO-5/6 and LET-767.The point of action of LET-767, the RNAi-targeted enzyme evaluated in this study, is indicated and highlighted with a green box.

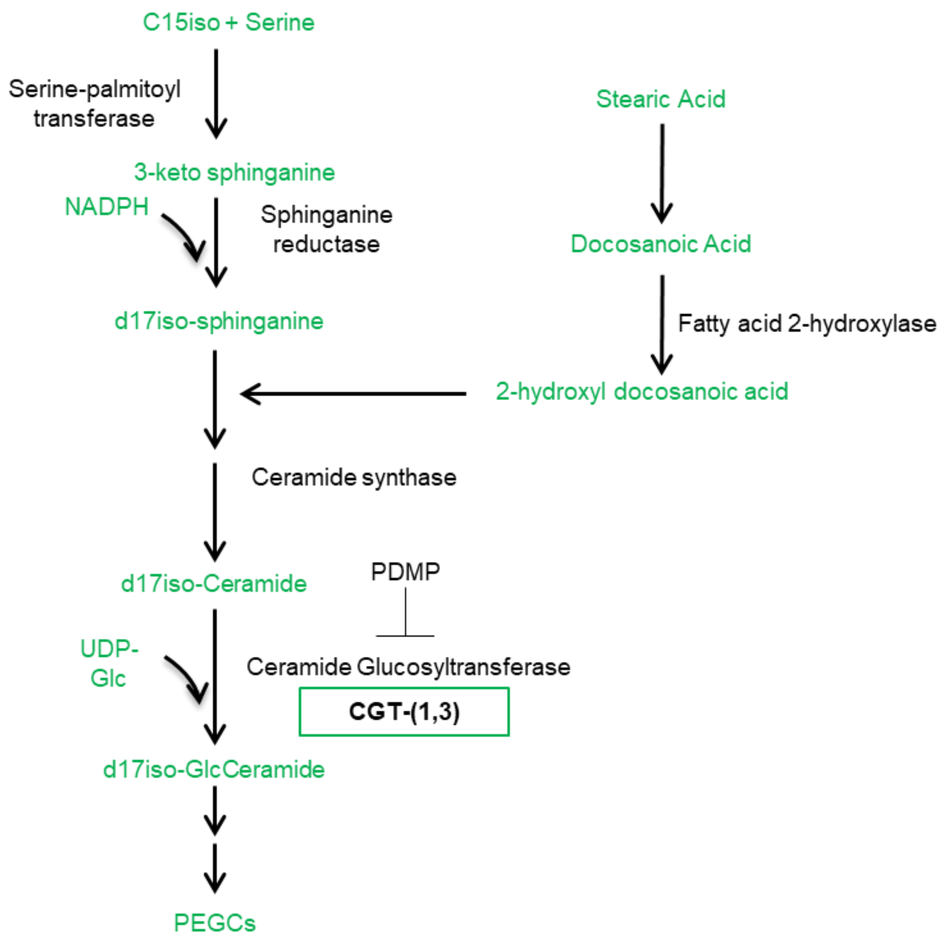

Supplementary Figure 6. Simplified biosynthesis pathway for d17iso-GlcCeramide from branched-chain fatty acid precursors in *C. elegans*. The enzymes involved in this pathway are depicted in a simplified scheme. Ceramide glucosyltransferase enzymes (CGT-1,3) are highlighted with a green box and the point of action of PDMP is indicated. Not shown are the enzymatic steps for the conversion of d17iso-sphinganine to d17iso-ceramide.

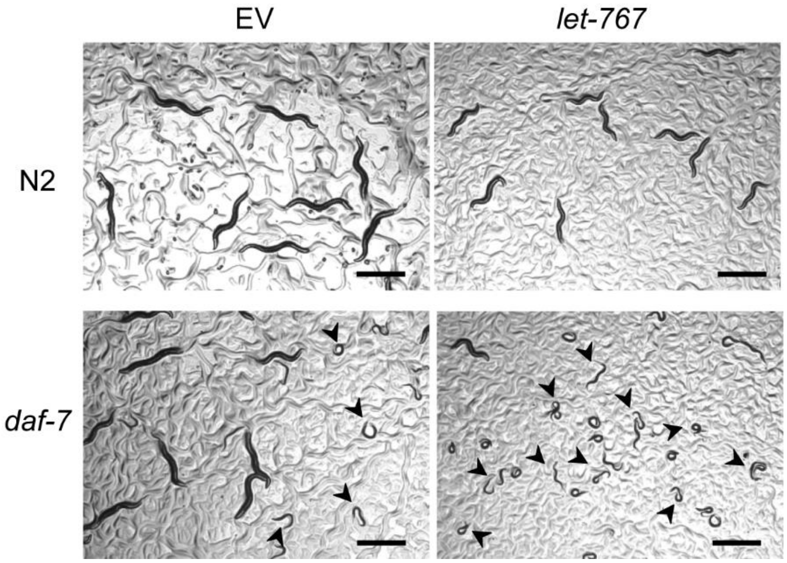

Supplementary Figure 7. Deficiency of iso-branched fatty acids leads to a high incidence of dauers in *daf-7* background mutants. *let-767* RNAi treated wild-type worms do not display a dauer formation phenotype. However, *daf-7* worms treated with *let-767* RNAi exhibit a strong dauer phenotype (indicated with arrowheads), in contrast to *daf-7* animals treated with an empty vector control. Representative images from at least three experiments. Scale bars, 0.5 mm.

Supplementary Table 1. LC-MS/MS detection and absolute quantification of 2-AG and AEA in lipid extracts of N2, *daf-7, fat-3* and *daf-7;fat-3* worms supplemented with methyl-AA (50 µM). *fat-3* and *daf-7;fat-3* mutants are capable of synthesizing 2-AG and AEA from the methyl-AA supplement.

	2-AG (pg/mg protein)	AEA (pg/mg protein)
N2	853.62 ± 294.00	19.74 ± 11.28
daf-7	1654.63 ± 745.22	17.93 ± 9.62
fat-3 + methyl-AA	964.58	8027.85
daf-7;fat-3 + methyl-AA	1210.5	10226.7

Supplementary Table 2. LC-MS/MS detection and absolute quantification of 2-AG in lipid extracts of synchronized N2 L4 worms grown for 72h at 20 ºC in the presence of cholesterol at the standard working concentration (13 µM) and under complete depletion of this sterol (0 µM).

[cholesterol]	[2-AG]
13 µM	2294.025±177.92 (pg/mg protein)
0 µM	4868.33± 438.08 (pg/mg protein) (*)

Measurements are informed as (mean values ± standard deviations). (*) indicates statistically significant difference with [cholesterol]=13 µM condition. t-test, p-value=0.002. The amount of independent experiments is n=3 for [cholesterol]=13 µM and n=2 for [cholesterol]= 0 µM.

Supplementary Table 3. *let-767* RNAi significantly decreases the mmBCFA content (15:0iso and 17:0iso) of *daf-7*.

FATTY ACID	Empty vector	*let-767* RNAi	p-value
14:0	2.28 ± 0.41	2.73 ± 0.41	0.246
15:0iso	6.83 ± 0.20	3.43 ± 0.27*	<0.001
15:0	0.08 ± 0.03	0.22 ± 0.1	0.074
16:0	4.30 ± 0.97	6.52 ± 2.49	0.224
17:0iso	4.38 ± 0.11	2.15 ± 0.37*	<0.001
18:0	5.19 ± 0.52	7.38 ± 0.33*	0.004
18:1 (n-9)	7.32 ± 1.19	3.41 ± 0.16*	0.005
18:1 (n-7)	19.22 ± 3.20	20.77 ± 3.29*	0.045
20:3 (n-6)	1.88 ± 0.24	1.99 ± 0.18	0.564
20:4 (n-6)	1.31 ± 0.40	1.10 ± 0.06	0.414
20:3 (n-3)	0.22 ± 0.08	0.10 ±0.02	0.070
20:4 (n-3)	1.81 ± 0.14	2.18 ± 0.56	0.308
20:5 (n-3)	6.32 ± 1.21	6.79 ± 1.23	0.657

Relative percentage values (mean ± standard deviation) of fatty acids measured by GC/MS analysis of *daf-7* worms grown on control conditions (empty vector) or *let-767* RNAi. (*) indicates significant difference with empty vector. t-test, α=0.05. The amount of independent experiments is n=3 for empty vector and *let-767* RNAi.

Supplementary Table 4. GC/MS analysis of fatty acid methyl-esters in *daf-7* mutants grown on empty vector or *let-767* RNAi in the absence or presence of 2-AG.

	EV	*let-767* RNAi	*let-767* RNAi+2-AG
SFA	13.42 ± 1.42	19.55 ± 2.74 (*)	17.95 ± 1.27
mmBCFA	12.55 ± 1.28	7.66 ± 1.73 (*)	6.92 ± 1.36
MUFA	34.88 ± 3.88	36.54 ± 4.36	34.28 ± 8.37
PUFA	23.42 ± 1.23	22.79 ± 1.69	25.68 ± 3.59
Cyclopropanes	15.74 ± 4.87	13.45 ± 3.30	15.18 ± 4.87

Endocannabinoid treatment does not revert the drop in mmBCFAs levels induced by *let-767* RNAi. The values represent the relative percentage of each fatty acid species (mean ± standard deviation). The number of independent experiments is n=3 for empty vector (EV) and *let-767* RNAi, and of n=2 for *let-767* RNAi+2-AG. SFA: straight chain saturated fatty acids; mmBCFA: mono-methyl branched chain fatty acids; MUFA: mono-unsaturated fatty acids; PUFA: polyunsaturated fatty acids. (*) indicates statistically significant difference with empty vector control. One-way analysis of variance, $p<0.05$. [2-AG]=50 µM.
